# Role of Copper Nanoparticles in the Thermal and Mechanical Properties of Expanded Graphite-Reinforced Epoxy Hybrids

**DOI:** 10.1021/acsomega.4c00394

**Published:** 2024-04-03

**Authors:** Hai-Long Cheng, Na Chu, Fan-Long Jin, Soo-Jin Park

**Affiliations:** † Department of Polymer Materials, Jilin Institute of Chemical Technology, Jilin 132022, People’s Republic of China; ‡ Department of Chemistry, Inha University, Michuhol-gu, Incheon 22212, South Korea

## Abstract

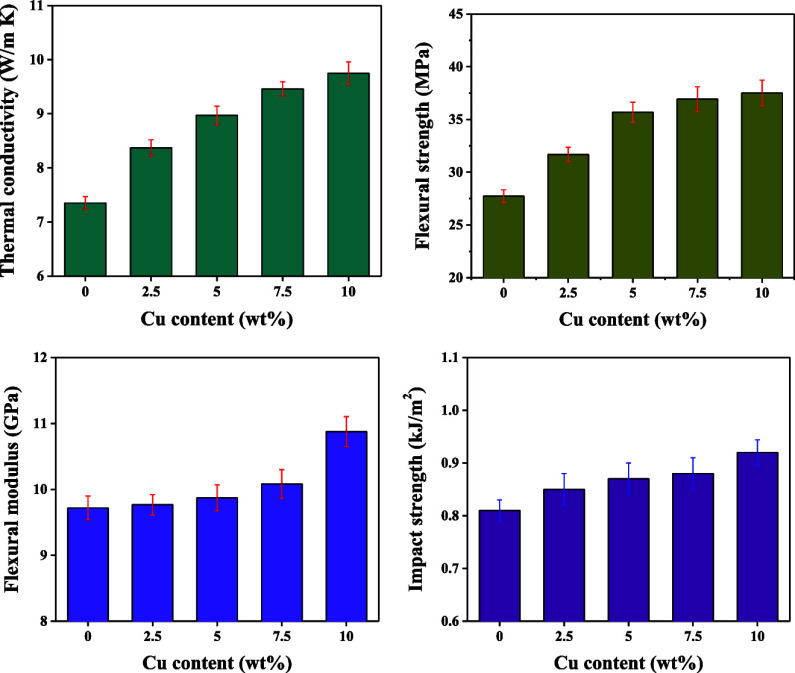

Epoxy resin is extensively applied in the electronics and electrical fields because of its outstanding comprehensive performance. However, the low thermal conductivity (TC) limits its application in thermal interface materials. In the present work, epoxy-based hybrid composites with high TC were prepared by using expanded graphite (EG) and copper (Cu) nanoparticles as thermally conductive hybrid fillers via hot blending and compression-curing processes. Additionally, the influence of the Cu content on the thermal properties, mechanical properties, and morphology of each epoxy/EG/Cu composite was investigated. According to the results, the epoxy/EG/Cu composite showed a maximum TC of 9.74 W/(m·K) at a fixed EG content of 60 wt % owing to the addition of 10 wt % Cu. After the addition of 10 wt % Cu, the flexural strength, flexural modulus, and impact strengths of epoxy/EG/Cu composites were improved from 27.9 MPa, 9.72 GPa, and 0.81 kJ/m^2^ to 37.5 MPa, 10.88 GPa, and 0.91 kJ/m^2^, respectively. Hence, this study offers a feasible strategy for the design of epoxy hybrid composites with excellent TC that can be applied to thermal interface materials.

## 1 Introduction

Epoxy resins are a high-performance thermosetting polymer and have a unique combination of characteristics, which include low shrinkage, high dimensional and thermal stability, excellent electrical insulation, high mechanical properties, high chemical resistance, and easy processing.^1−5^ Diglycidyl ether of bisphenol A (DGEBA) epoxy resin is created when epichlorohydrin and bisphenol A react with a basic catalyst. DGEBA contains benzene, methyl, and hydroxyl groups in the main molecular chain, which exhibits excellent comprehensive properties.^6,7^ Consequently, epoxy resin is extensively applied in the electronics and electrical fields. However, owing to its low crystallinity, epoxy resin exhibits low thermal conductivity (TC), which limits its application in thermal interface materials. Therefore, it has become crucial to improve the TC of epoxy resin.^8−11^

Typically, thermally conductive polymer-based composites are fabricated by dispersing high-thermal-conductive fillers, including carbon-based fillers, metals, and inorganic particles, into the polymer matrix. Carbon-based fillers include graphite, carbon fibers, graphene, and carbon nanotubes (CNTs), each of which shows high TC and excellent mechanical performance.^12−16^ Particularly, graphite has excellent thermal and electrical conductivities, a high self-lubrication ability, resistance to both high and low temperatures, good corrosion resistance, and good chemical stability. Additionally, expanded graphite (EG) can be synthesized easily and affordably by reacting natural flake graphite with an interlaminar agent and then expanding the graphite sheets at a high temperature; this increases the specific surface area of the graphite sheets while retaining the above-mentioned advantageous properties of graphite. After the expansion process, a portion of the graphite reacts with the oxidation phase to generate functional groups on the EG surface, thereby providing it with good mechanical properties and potential modification sites.^17−21^

Alternatively, metal fillers can be used to fabricate polymer-based composites with high TC and electrical conductivity, as well as high dielectric constants. When copper (Cu) crystals are ideally arranged, a high TC of 397 W/(m·K) can be obtained. Notably, this provides TC similar to that of silver (Ag) but at a much lower cost. As a result, Cu is more commonly used than Ag as a highly thermally conductive filler in various industries.^22−25^

A single type of thermally conductive filler often needs to be added in significant quantities to achieve high TC in a polymer-based composite. This makes the preparation process difficult and also causes agglomeration of the filler and the formation of bubbles in the polymer matrix, thereby dramatically reducing the mechanical performance of the resulting composites.^26−28^ Therefore, several researchers have reported the combined use of a variety of thermally conductive hybrid fillers to improve the TC of epoxy resin.^29−34^ For example, Zhang et al. studied the TC of epoxy composites containing Cu nanoparticles and multiwalled CNTs (MWCNTs).^29^ They reported that the TC of a composite containing 15 wt % MWCNTs and 34 wt % Cu nanoparticles was 0.58 W/(m·K), which is 3.2 times greater than that of the pure epoxy resin sample. Isarn et al. investigated the TC of epoxy coatings using EG and boron nitride hybrid fillers.^30^ Their results showed that the TC of a composite with 72.5 wt % hybrid fillers was 2.08 W/(m·K). Kumar et al. used hand layup and mechanical mixing to synthesize EG and graphene-reinforced epoxy resin-based composites.^31^ Their results revealed that a composite containing 35 wt % hybrid fillers exhibited a TC of 3.6 W/(m·K). Furthermore, the same authors investigated the TC of hybrid epoxy composites containing EG and silver flake hybrids; they obtained a TC of 3.42 W/(m·K) by adding 30 wt % EG/Ag.^32^ Liu et al. prepared reduced graphene oxide-encapsulated Cu sphere (Cu@rGO) hybrids and used them as fillers to improve the TC of epoxy resin.^33^ The TC of the resulting epoxy composites reached 7 W/(m·K) at 80 wt % hybrids. In another study, Yim and Park enhanced the TC by adding hybrid fillers consisting of silver-plated EG, graphite, and Cu powder.^34^ The above results indicated that the research focused on the use of hybrid fillers to improve the TC of the epoxy resin.

This work aims to design epoxy hybrid composites with excellent TC and apply them to thermal interface materials. In this study, a feasible method for improving the TC of DGEBA was developed by employing EG and Cu nanoparticles as thermally conductive hybrid fillers. Additionally, the effects of the Cu content on the thermal properties, mechanical properties, and morphologies of the DGEBA/EG/Cu composites were studied. After adding EG and Cu nanoparticles to the DGEBA matrix, the nanoparticles penetrated the graphite sheets’ pores to form a so-called lamellar-sphere-lamellar structure, thereby reducing the interfacial thermal resistance. At the same time, the nanoparticles formed a thermal bridge between the graphite sheets, thereby promoting the construction of a continuous thermally conductive pathway, which significantly improved the TC of the resulting composites. In the present work, the problem of low TC of the epoxy resin was solved by preparing DGEBA/EG/Cu hybrid composites.

## 2 Experimental Section

### 2.1 Materials

**Figure 1:**
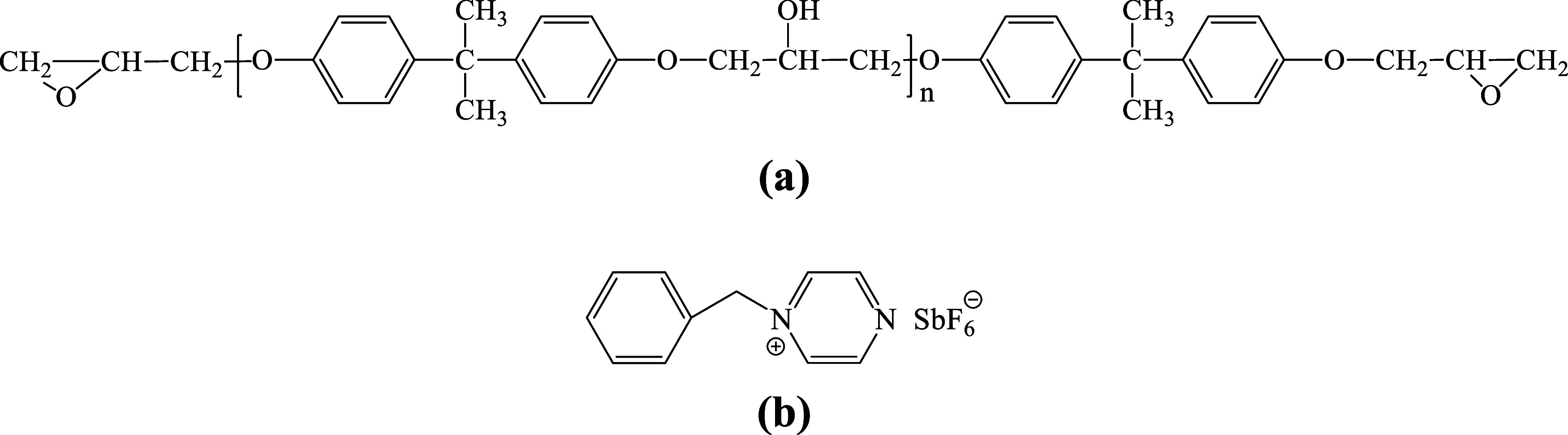
Chemical structures of (a) DGEBA and (b) BPH.

**Figure 2:**
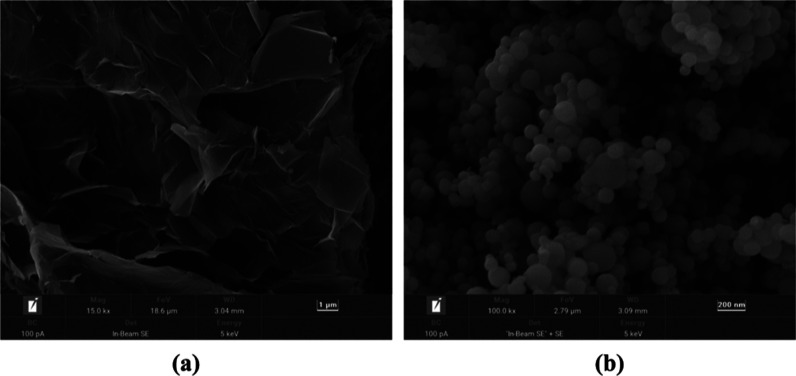
SEM micrographs of the (a) EG (15,000×; scale bar = 1 μm) and the (b) Cu nanoparticles (100,000×; scale bar = 200 nm).

DGEBA (184–195 g/mol) was obtained from Nantong Xingchen Synthetic Material Co., Ltd. Following a previously reported procedure, a thermally latent initiator of the DGEBA epoxy resin *N*-benzylpyrazinium hexafluoroantimonate (BPH) was synthesized.^35^ The structures of the epoxy resin and thermally latent initiator are presented in Figure 1. The EG was supplied by Jiangxi Shuobang New Material Technology Co., Ltd. The Cu nanoparticles (a particle size of 50 nm) were supplied by Zhongxin New Materials. The high-resolution scanning electron microscopy (HR-SEM) images of the as-supplied EG and Cu nanoparticles are shown in Figure 2.

### 2.2 Preparation of the DGEBA/EG/Cu Composite Samples

**Figure 3:**
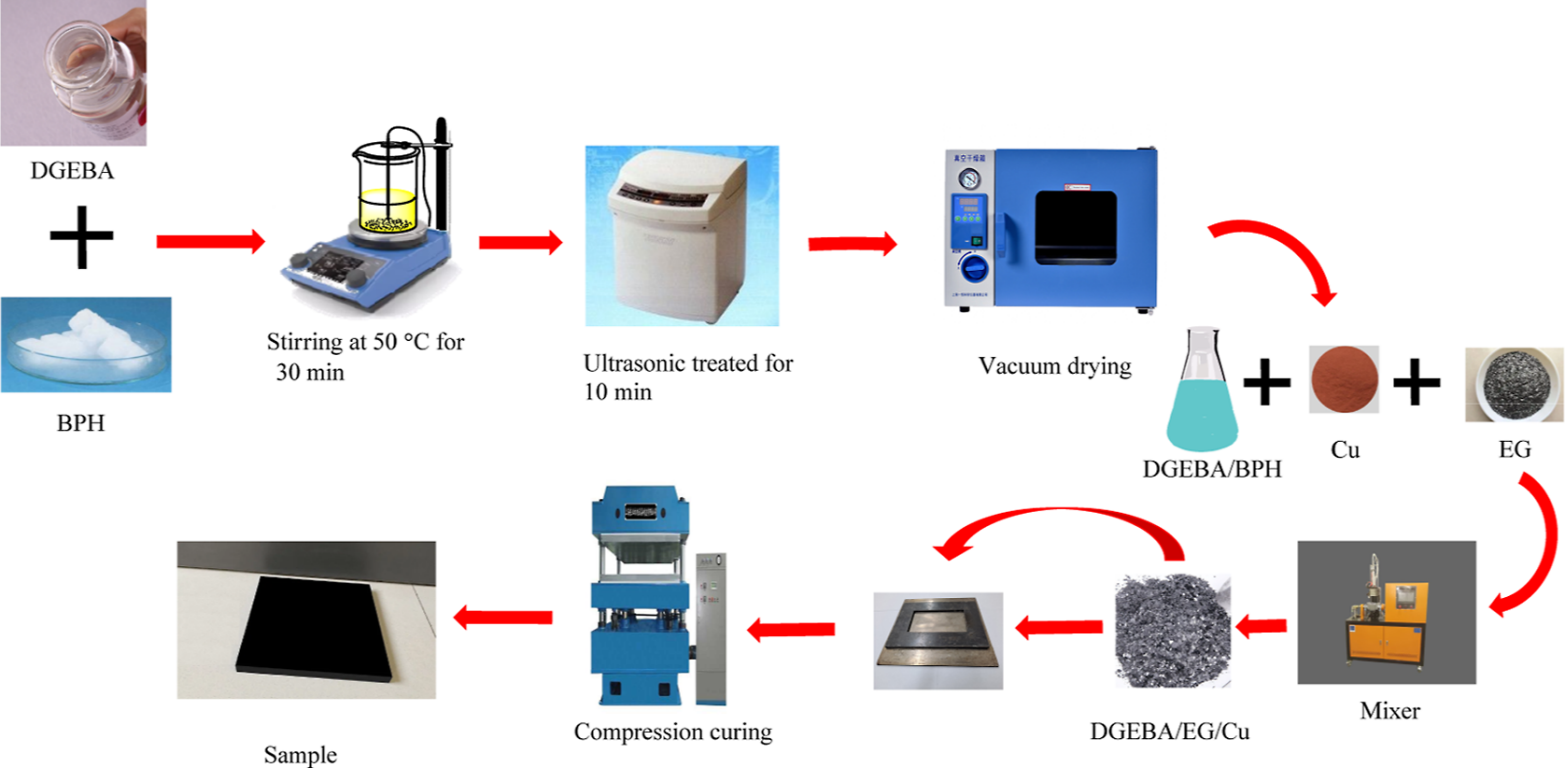
Schematic diagram showing the preparation of the DGEBA/EG/Cu composites.

A schematic illustration of the DGEBA/EG/Cu composite synthesis is shown in Figure 3. The EG/DGEBA ratio was set at 60:40, and the Cu content was 0–10 wt %. DGEBA and BPH were blended via mechanical stirring at 50 °C for 30 min, ultrasonicated for 10 min, and then degassed under reduced pressure. Subsequently, the desired amounts of EG and Cu were added to the DGEBA/BPH system and mixed at 80 °C for 30 min. The mixture was then injected into a mold that had been preheated. The composite samples were compression-cured at 120, 150, and 200 °C under a pressure of 5 MPa for 1 h.

### 2.3 Characterization and Measurements

**Figure 4:**
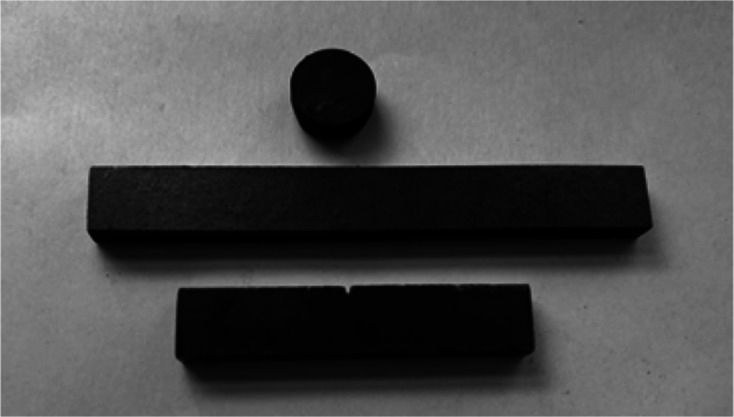
Pictures of various specimens for TC, flexural properties, and impact strength tests.

The morphologies of the EG and Cu nanoparticles were observed by HR-SEM (JSM-7610F Plus). The TC of the 5 × 10 × 30 mm^3^ composite samples was characterized by a TC tester (WNK-100) according to the GB/T 10294-2008 standard, and the average of the five experimental values was used as the result. The thermal stabilities of the samples were evaluated using thermogravimetric analysis (TGA; TA Instruments, Q50) at 30–800 °C, 10 °C/min, and a N_2_ atmosphere. The resulting TGA curves were used to calculate various thermal stability factors, including the temperatures at which a 5 and 10% weight loss occurred (*T*_5%_ and *T*_10%_, respectively) and the amount of char formation at 800 °C; this was carried out using previously reported procedures.^36,37^ The flexural performance of the samples was investigated via a mechanical testing apparatus (WDW 3010) according to the GB/T 9341-2008 standard. The sample size was 4 × 10 × 80 mm^3^, the span length was 64 mm, and the cross-head speed was 2 mm/min. The flexural strength (σ_f_) and elastic modulus (*E*_b_) values were determined using eqs 1 and 2, respectively
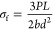
1
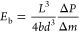
2where *P* is the applied load, *L* the span length, *b* the specimen’s width, *d* the specimen’s thickness, Δ*P* the change in force in the linear portion of the load-deflection curve, and Δ*m* is the corresponding change in deflection. Five experimental values were averaged to obtain the flexural strength and modulus values. Additionally, the impact strengths of the samples were investigated by an Izod impact tester (TP04G-AS1) according to the GB/T 1843-2008 standard. The impact strength was calculated by averaging the five experimental values. Figure 4 shows pictures of various specimens for TC, flexural properties, and impact strength tests. After the impact strength tests, the morphologies of the composites were investigated using field-emission SEM (FE-SEM, Hitachi, S-4800). The fracture surface of the sample was cut into 1 mm slices, and the surface was sprayed with gold.

## 3 Results and Discussion

### 3.1 Thermal Conductivity

**Figure 5:**
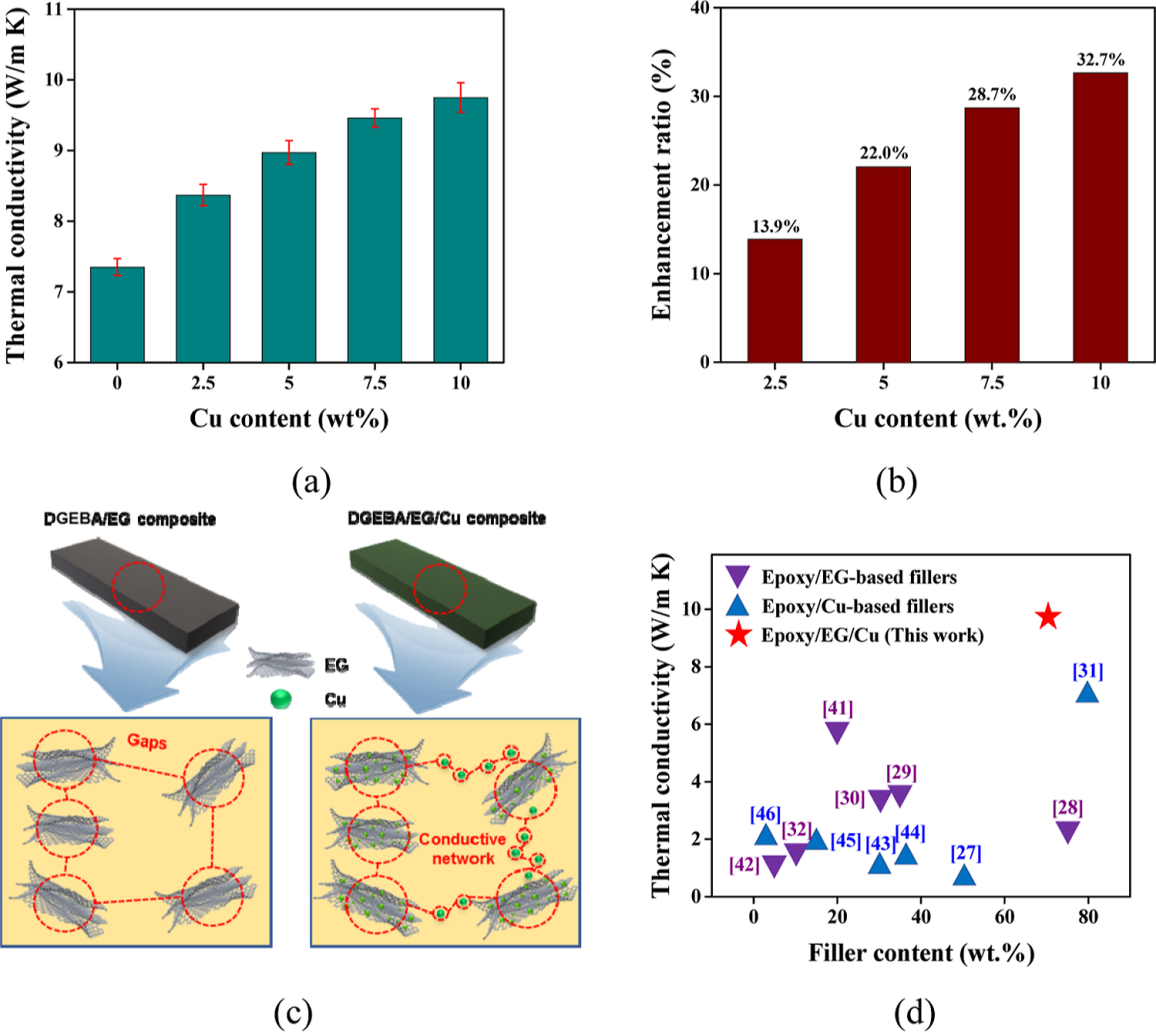
(a,b) TC (a) and TC enhancement ratio (b) of the DGEBA/EG/Cu composites with various Cu contents. (c) Schematic diagram showing the TC mechanisms of the DGEBA/EG (left-hand) and DGEBA/EG/Cu (right-hand) composites. (d) Comparison between the TC of the as-prepared DGEBA/EG/Cu composite and those of previously reported epoxy/EG-based composites and epoxy/Cu-based composites.

Figure 5a illustrates the influence of various Cu contents on the TC of the DGEBA/EG/Cu composite. Here, the TC of the composite improved from 7.35 to 9.74 W/(m·K) as the Cu content increased from 0 to 10 wt %. Furthermore, the TC enhancement ratios of the DGEBA/EG/Cu composites with various Cu contents are presented in Figure 5b, where the enhancement in TC is seen to be more obvious when the Cu content is low. This can be attributed to the aggregation of some Cu nanoparticles at a high Cu content, which affects the formation of the thermally conductive network. Kumar et al. achieved similar results using EG and graphene as hybrid fillers; the TC of the resulting composite reached 3.6 W/(m·K).^31^

The mechanism behind the improved TC is illustrated schematically in Figure 5c. As demonstrated in previous studies, the lamellar-structured EG has a high TC and forms a partial thermal conduction pathway in the DGEBA/EG composite (left-hand panel, Figure 5c);^38−41^ however, there are numerous pores within the graphite sheets and large gaps between the graphene sheets, each of which increases the thermal resistance of the interface. With the addition of Cu nanoparticles, the nanoparticles penetrate the pores of the graphite sheets to form a so-called lamellar-sphere-lamellar structure, thereby decreasing the interfacial thermal resistance while also forming a thermal bridge between the graphite sheets; this promotes the construction of a continuous thermally conductive pathway (right-hand panel, Figure 5c).^35,42^ Therefore, the TC of the DGEBA/EG/Cu composite can be increased by the addition of Cu nanoparticles. Consequently, as demonstrated in Figure 5d, the as-prepared epoxy/EG/Cu composites show a high TC of up to 9.74 W/(m·K), which is superior to that of previously reported epoxy/EG-filler-based composites and epoxy/copper-filler-based composites, where the highest reported TC was only 7 W/(m·K), even under a high loading of hybrid fillers.^28−34,43−47^ The DGEBA hybrid composite prepared in this study has excellent TC, but the content of hybrid fillers is relatively large. Future research will need to focus on further improving the TC of epoxy-based composites while maintaining a low hybrid filler content.

### 3.2 Thermal Properties

**Figure 6:**
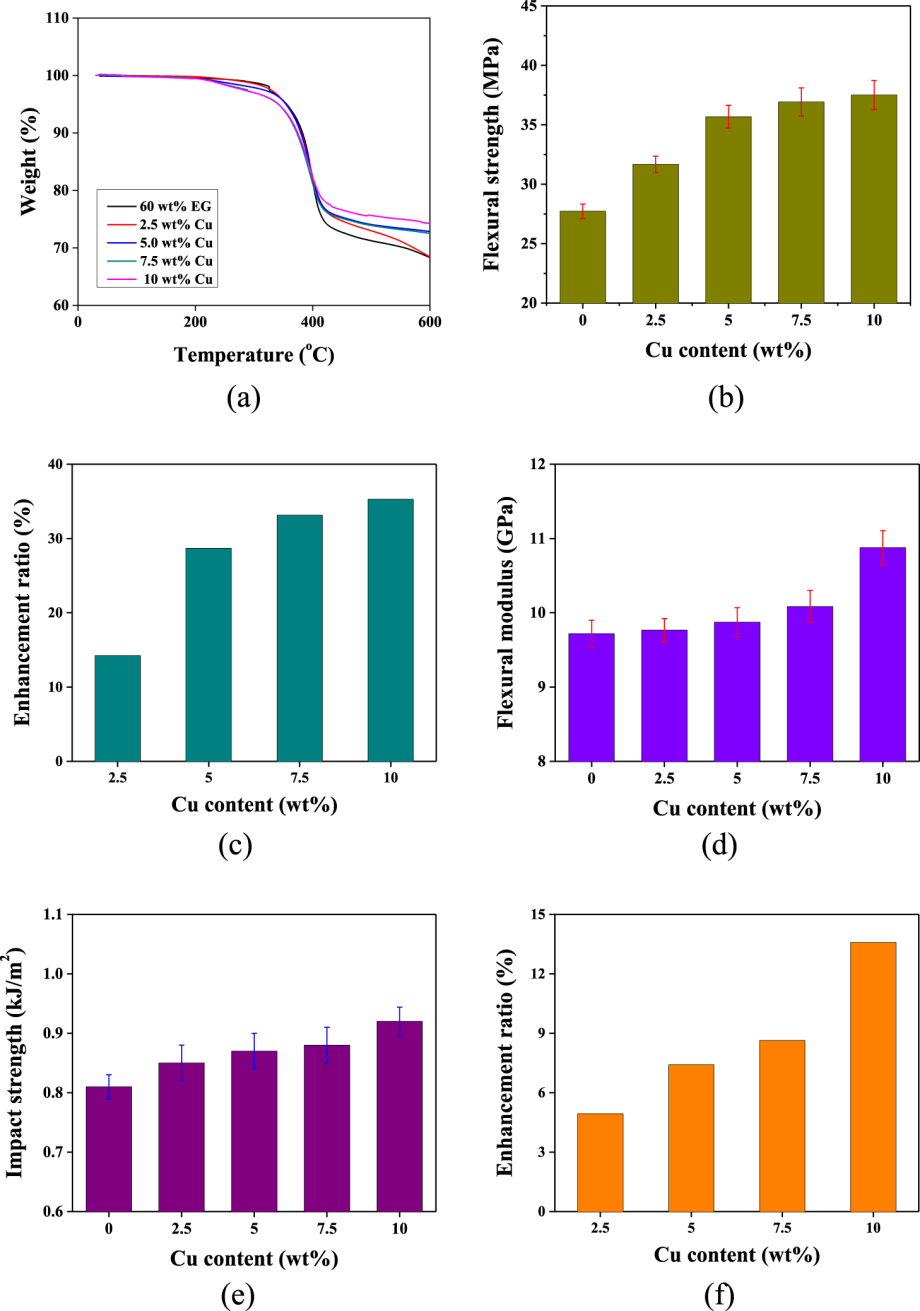
TGA thermograms (a), flexural strengths (b), flexural strength enhancement ratios (c), flexural moduli (d), impact strengths (e), and impact strength enhancement ratios (f) of the DGEBA/EG/Cu composites according to the Cu content.

The thermal stabilities of the DGEBA/EG/Cu composites with various Cu contents are shown in the TGA results in Figure 6a and the corresponding (calculated) thermal stability factors in Table 1. The *T*_5%_ and *T*_10%_ values of the DGEBA/EG/Cu composites decreased slightly from 355.7 and 382.8 °C in the absence of Cu to 341.1 and 374.6 °C by adding 10 wt % Cu, representing a decrease of 14.6 and 8.2 °C, respectively. This is attributable to the good TC of the Cu nanoparticles. When the sample was heated, the temperature was higher around the Cu nanoparticles than in other areas of the sample; this accelerated the thermal degradation of the polymer around the Cu nanoparticles.^48^ Additionally, the char amount at 800 °C increased as the Cu nanoparticles were added; this was due to the large residual mass of the Cu nanoparticles.

**Table 1:** Factors for Thermal Stability of DGEBA/EG/Cu Composites

Cu amount (wt %)	*T*_5%_ (°C)	*T*_10%_ (°C)	char at 800 °C (%)
0	355.7	382.8	68.3
2.5	354.2	380.7	68.4
5.0	354.8	379.8	72.8
7.5	342.0	372.6	72.5
10	341.1	374.6	74.2

### 3.3 Flexural Properties

The flexural strengths and enhancement ratios of the composites with various Cu contents are presented in Figure 6b,c, respectively. Here, the flexural strength increased by 34%, from 27.9 MPa in the absence of Cu to 37.5 MPa in the presence of 10 wt % Cu. This is because of the application of an external force to the DGEBA/EG composite, which caused the energy to be absorbed by crack formation in the graphite sheets dispersed in the DGEBA matrix until the deformation exceeded its limit. However, upon the addition of Cu nanoparticles, the nanoparticles can penetrate the pores of the graphite sheets and generate numerous microcracks in the DGEBA matrix, increasing its ability to absorb energy and resist deformation. Additionally, the Cu nanoparticles have a certain affinity for oxygen and improve the interfacial interactions with the DGEBA matrix, thereby increasing the flexural strength of the composites.^49,50^ The effects of various Cu contents on the flexural moduli of the composites are revealed in Figure 6d. The flexural modulus increased by 13%, from 9.72 GPa in the absence of Cu to 10.88 GPa in the presence of 10 wt % Cu. This increase occurred because the addition of Cu nanoparticles increased the stiffness of the composite, thereby increasing its flexural modulus.^51^

### 3.4 Impact Strength

The impact strengths and enhancement ratios of the composites with various Cu contents are shown in Figure 6e,f, respectively. Here, the impact strength increased by 13.6%, from 0.81 kJ/m^2^ in the absence of Cu to 0.91 kJ/m^2^ in the presence of 10 wt % Cu. This is because the Cu nanoparticles can disperse throughout the DGEBA/EG composite, including inside the pores of the graphite sheets, and they can generate numerous microcracks in the DGEBA matrix, thereby absorbing any external impact energy and, hence, improving the impact strength of the DGEBA/EG/Cu composite.^52^

### 3.5 Morphology

**Figure 7:**
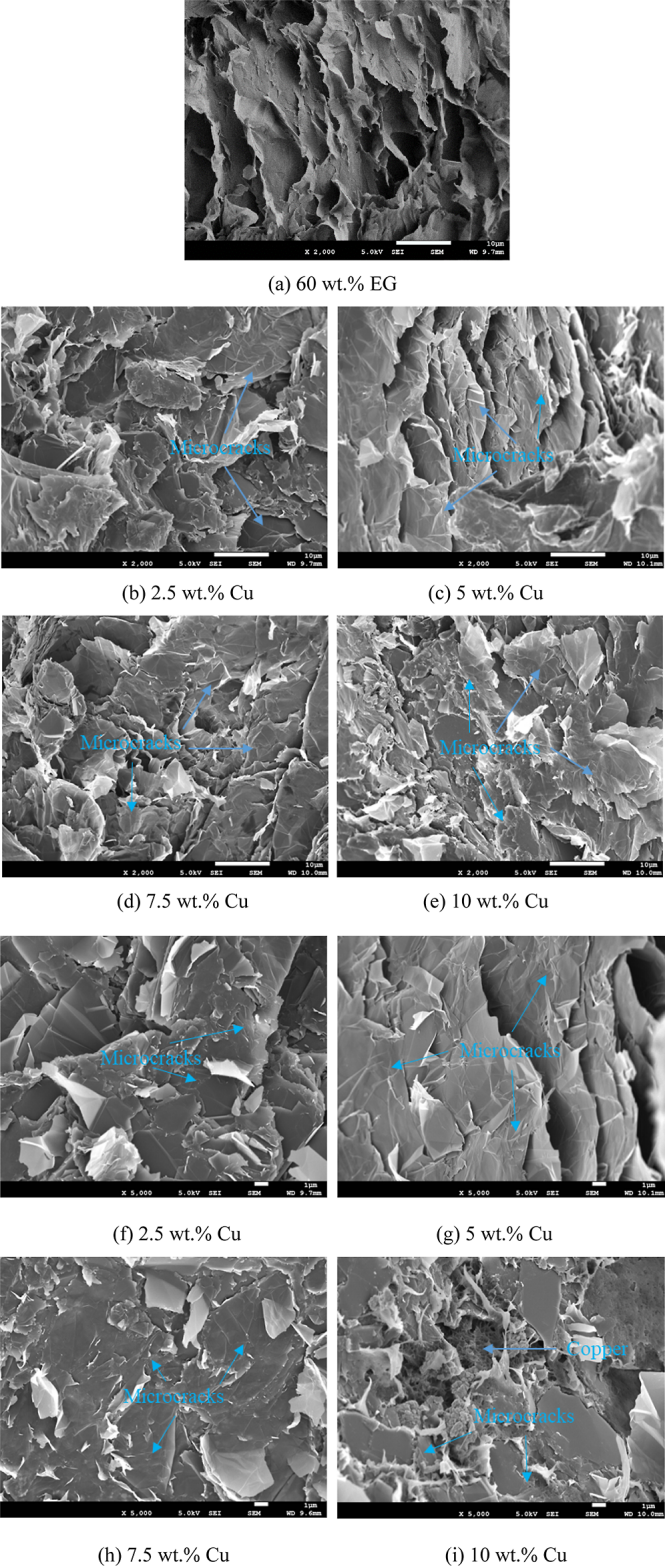
SEM micrographs of the DGEBA/EG/Cu composites with (a) 60 wt % EG only, (b) 60 wt % EG + 2.5 wt % Cu, (c) 60 wt % EG + 5 wt % Cu, (d) 60 wt % EG + 7.5 wt % Cu, (e) 60 wt % EG + 10 wt % Cu (2000×; scale bar = 10 μm), (f) 60 wt % EG + 2.5 wt % Cu, (g) 60 wt % EG + 5 wt % Cu, (h) 60 wt % EG + 7.5 wt % Cu, and (i) 60 wt % EG + 10 wt % Cu (5000×; scale bar = 1 μm).

The morphologies of the different DGEBA/EG/Cu composites after impact strength tests are revealed in the SEM images in Figure 7. Here, the DGEBA/EG composite exhibits sheet-like blocks that peel away from the fractured surface under an external impact force (Figure 7a), thus accounting for the low impact strength.^53^ Nevertheless, the EG sheets form a local thermally conductive pathway that endows the DGEBA/EG composite with relatively high TC. As various quantities of Cu nanoparticles were added, the number of sheet-like blocks increased, and numerous microcracks appeared in the DGEBA matrix (Figure 7b–i); these microcracks absorb more energy upon the application of an external impact force.^54^ Meanwhile, the DGEBA/EG/Cu composites maintain sheet-like blocks that form a local thermally conductive pathway. Additionally, as Cu nanoparticles were added, the Cu nanoparticles clustered between the graphite sheets (high-resolution image in Figure 7i), where they can act as thermal bridges to form a thermal conduction pathway in the DGEBA matrix, thus improving the TC of the DGEBA/EG/Cu composites.^30,35,55^

## 4 Conclusions

In this study, the TC of DGEBA was improved by adding EG and various amounts of Cu via hot blending and compression-curing processes. The TC and stabilities of the various composites were investigated along with their flexural properties, impact strengths, and fracture-surface morphologies. The TC of the DGEBA/EG/Cu composites improved from 7.35 to 9.74 W/(m·K) (a 32.5% increase) with an increase in the Cu content from 0 to 10 wt %, which is because Cu nanoparticles can penetrate the pores of the graphite sheets to form a so-called lamellar-sphere-lamellar structure and act as a thermal bridge between neighboring graphite sheets to construct a continuous thermally conductive pathway. The thermal stabilities of the composites decreased with the addition of Cu, while the flexural strength, flexural modulus, and impact strength increased by 34, 13, and 13.6%, respectively. The SEM images revealed that the Cu nanoparticles between the graphite sheets act as thermal bridges to form a continuous thermally conductive pathway in the DGEBA/EG/Cu composites. Hence, the present work provides a feasible method for preparing epoxy composites with excellent TC.
